# Activation of uroepithelial 5-HT_4_R inhibits mechanosensory activity of murine bladder afferent nerves

**DOI:** 10.3389/fphys.2022.990178

**Published:** 2022-09-13

**Authors:** Yu Lu, Jie Li, Li Dong, Ping Luo, Guohua Zhang, Weifang Rong

**Affiliations:** Department of Anatomy and Physiology, Shanghai Jiao Tong University School of Medicine, Shanghai, China

**Keywords:** uroepithelium, 5-HT4R, micturition, overactive bladder, bladder afferent nerve

## Abstract

Serotonin (5-HT) is known to act *via* multiple 5-HT receptors at spinal and supraspinal levels to regulate micturition. However, the contribution of peripheral 5-HT and its receptors in bladder physiology and pathology is not very well understood, despite evidence showing expression of multiple 5-HT receptors in the bladder wall and 5-HT may activate bladder afferent nerves. The current study was designed to investigate the possible role of 5-HT_4_R in modulation of the sensitivity of bladder afferents to bladder filling. Immunofluorescent staining showed abundant 5-HT_4_R immunoreactivity largely confined to the uroepithelium in wild type (WT) but not 5-HT_4_R^−/−^ mice. In the *ex vivo* bladder-pelvic nerve preparation, intravesical application of the 5-HT_4_R agonist RS67333 (1–30 μm) caused concentration-dependent decreases of the pelvic nerve response to bladder filling. Such effect was not observed in the presence of 5-HT_4_R antagonist GR125487 or in 5-HT_4_R^−/−^ preparations. A cohort of 5-HT_4_R^−/−^ and WT control mice were treated with intraperitoneal injections of cyclophosphamide (CYP) (75 mg/kg, three times at 2 days interval) to induce chronic cystitis. Void spot analysis showed that CYP-treated 5-HT_4_R^−/−^ mice urinated more frequently than their CYP-treated WT counterparts. Concomitantly, bladder afferents of CYP-treated 5-HT_4_R^−/−^ mice displayed exaggerated sensitivity to bladder filling in comparison with the CYP-treated WT controls. These data suggest that 5-HT_4_R expressed on uroepithelial cells plays an inhibitory role in mechanosensory transduction in the bladder. Loss of 5-HT_4_R-mediated inhibition may enhance bladder afferent sensitivity and exacerbate bladder overactivity in pathological conditions. We propose that 5-HT_4_R agonists might be exploited for the treatment of overactive and painful bladder symptoms.

## Introduction

Serotonin (5-hydroxytryptamine, 5-HT) is an important neurotransmitter, which has been implicated in control of genitourinary function besides its well-known role in regulation of mood, pain and body temperature (Berger et al., 2009). Within the central nervous system (CNS), 5-HT acts via different 5-HT receptor (5-HTR) subtypes at spinal and supraspinal sites to regulate autonomic neural input to the bladder and somatic neural input to the external urinary sphincter. Activity in the CNS serotonergic pathway generally enhances urine storage by facilitating the vesical sympathetic reflex pathway and inhibiting the parasympathetic voiding pathway, a rationale for the use of combined serotonin-norepinephrine reuptake inhibitors in the treatment of stress incontinence (Ariman et al., 2021).

The great majority (∼95%) of the total body content of 5-HT is synthesized in the periphery (Banskota et al., 2019). Enterochromaffin (EC) cells and mast cells are well-known peripheral sources of 5-HT. Recently, 5-HT-synthesizing neuroendocrine cells have also been localized in the urethral epithelium (Kullmann et al., 2018). 5-HT released from those cells can act locally, or enter the circulation (largely stored in platelets) to act remotely. Thus, 5-HT released from EC cells in response to luminal contents not only plays a major role in gastrointestinal motility (Gershon and Michael, 2013), but also influences bone metabolism (Ducy, 2011), and glucose homeostasis (Martin et al., 2017). However, how peripheral 5-HT affects urinary bladder function in normal and disease states is not very well understood. Understanding how peripheral 5-HT affects urinary bladder function may be of clinical relevance since serum 5-HT level has been associated with bladder symptoms (Okamoto et al., 2021).

The mechanosensory activity of the pelvic bladder afferents during bladder filling initiates the micturition reflex. Dysregulation of the bladder afferent mechanosensitivity may lead to bladder dysfunctions and pain. The uroepithelium is known to express mechanosensitive ion channels, such as Piezo1/2 and TRPV4, and may release excitatory and inhibitory mediators to modulate the afferent sensitivity in response to bladder filling (Birder and Andersson, 2013; Wu et al., 2021). 5-HT also appears to modulate bladder afferent sensitivity as well as detrusor contractility. Intravesical application of 5-HT caused concentration-dependent increase of bladder afferent discharge (Sun et al., 2010). Konthapakdee et al. reported that in the *ex vivo* bladder-pelvic nerve preparation, bath application of 5-HT may activate bladder afferent fibers, which was primarily mediated by 5-HT_3_R on afferent neurons. Furthermore, they detected transcripts of multiple 5-HT receptors in murine urinary bladder, including 5-HT1A, 5-HT1B, 5-HT1D, 5-HT2A, 5-HT2B, 5-HT4, 5-HT6, and 5-HT7 receptors (Konthapakdee et al., 2019). Earlier pharmacological studies have demonstrated that 5-HT_4_R agonists may either facilitate or inhibit detrusor contraction in different species (Messori et al., 1995; Candura et al., 1996). These results indicate that peripheral 5-HT may affect urinary bladder function. Nevertheless, the exact localization and function of 5-HT receptors in the urinary bladder are yet to be determined.

Given the above, the current study has been designed to investigate the localization of 5-HT_4_R in the murine urinary bladder through immunohistochemistry. Effects of intravesical 5-HT_4_R agonist on mechanosensory activity was studied in the *ex vivo* bladder-pelvic nerve preparation, using wild type (WT) and 5-HT_4_R^−/−^ mice. To understand the pathological relevance of uroepithelial 5-HT_4_R-mediated signaling, urination frequency and bladder afferent mechanosensitivity were compared in WT and 5-HT_4_R^−/−^ mice subjected to cyclophosphamide (CYP)-induced cystitis. Our results suggest that uroepithelial 5-HT_4_R mediates an inhibitory effect on bladder mechanosensation.

## Materials and methods

### Animals

Adult female 5-HT_4_R^−/−^ (8–10 W) and age-matched WT C57/BL6 mice were used in this study. The 5-HT_4_R^−/−^ mice were generated in Cyagen Biosciences Inc. A 1000 bp sequence of exon five fragments of the 5-HT_4_R gene (GenBank accession number: NM_008313.4; Ensembl: ENSMUSG00000026322) was deleted though CRISPR/Cas9. A line of 5-HT_4_R^−/−^ mice were bred in the Animal Facility of Shanghai Jiao Tong University School of Medicine, which also provided the WT C57/BL6 mice. All animals were housed in a temperature-controlled room (25°C) with a 12 h light/dark cycle and free access to food and water. All experiments were conducted in compliance with the governmental regulations on the use of experimental animals and had been approved by the Ethics Committee of Shanghai Jiao Tong University School of Medicine.

### Immunofluorescence

Mice were euthanized by an overdose of sodium pentobarbital and were immediately perfused with phosphate-buffered saline (PBS) followed by 4% paraformaldehyde (PFA). The bladder was dissected out and postfixed in 4% PFA. After fixation, the tissue was dehydrated in 30% sucrose solution for 24 h and embedded in OCT on the dry ice. For immunofluorescence, the bladder was cut into 15 μm thick sections. Sections were blocked with 10% normal donkey serum (NDS) and 1% Triton X-100 in PBS for 2 h and were then incubated with the primary antibodies (Anti-5-HT_4_R, 1:200, #AB60359, Abcam, Rabbit polyclonal; Anti-5-HT, 1:200, #GTX31099, GeneTex, Goat polyclonal) diluted in PBS containing 10% NDS at 4°C overnight. Sections were washed 4 times with PBS, then incubated with the secondary antibodies (Alexa Fluor 488-conjugated donkey anti-rabbit IgG, 1:1,000, #A21206, Invitrogen; Alexa Fluor 568-conjugated donkey anti-goat IgG, 1:1,000, #A11057, Invitrogen) 2 h at room temperature, and the nuclei were stained with DAPI (1:1,000, #PA5-62248, Thermo Fisher Scientific). Sections were examined with a LEICA DM 2500 microscope equipped for epifluorescence.

### Afferent nerve recording in the *ex vivo* bladder-pelvic nerve preparation

Mice were deeply anaesthetized with pentobarbital (80 mg/kg, i. p.), and the whole urinary tract attached to the surrounding tissues was dissected from the animal and placed in a recording chamber. The tissue was continuously perfused with oxygenated (95% O_2_–5% CO_2_) Krebs solution (contents, mM: NaCl, 120; KCl, 5.9; NaH_2_PO_4_, 1.2; MgSO_4_, 1.2; NaHCO_3_, 15.4; CaCl_2_, 2.5; glucose, 11.5). The bilateral ureters were tied with sutures. A two-way PE10 cannula was inserted into the bladder dome to enable infusion of fluid and recording of intravesical pressure. Another catheter was inserted into the bladder through the urethra to allow evacuation of fluid. This dual catheterization (dome and urethra) allowed for continuous intravesical instillation and rapid washout of drugs applied. The pelvic nerves emanating from the spinal cord were carefully dissected. A branch of the pelvic nerve was recorded using a suction electrode. Electrical activity was picked up by a Neurolog headstage (Neurolog NL900D, Digitimer, United Kingdom), amplified (NL104) and filtered (band pass 300–3,000 Hz). The intravesical pressure and the neural signal were fed into a computer via the Micro1401 interface (Cambridge Electronic Design, United Kingdom). Signals were monitored and analyzed using spike two software (version 3.2, Cambridge Electronic Design, United Kingdom). Afferent sensitivity to gradual filling of the bladder was assessed by infusion with saline at a rate of 0.3 ml/min. This was repeated at 15 min intervals until the response stabilized. To study drug effects on bladder afferent mechanosensitivity, the bladder was infused with saline containing required concentration of the drug (RS67333, GR125487 or both).

### Cyclophosphamide-induced cystitis

Mice were anesthetized with isoflurane (2%) and received intraperitoneal (i.p.) injections of cyclophosphamide (CYP, #C0768, Sigma) to induce urinary bladder inflammation. Each mouse received three injections of CYP (75 mg/kg i. p.) with an interval of 2 days (Girard et al., 2012). Control mice received saline treatment. Void spot assay and bladder afferent nerve recording were made on the eighth day.

### Void spot assay

Urination frequency was studied using the void spot assay (VSA) (Yu et al., 2014). Individual mice were gently placed into a mouse metabolic cage with precut rectangular-shaped filter paper taped to the bottom. Mice were provided with standard chow for the duration of the assay, but water was withheld during the testing period, which last for 3 h. At the end of the 3 h observation period, the filter paper was retrieved from the cage, and urine spots on the filter paper were imaged using ultraviolet light on a trans illuminator. Considering urinary frequency of C57BL/6J mice varies with respect to the time of day, VSA experiments were conducted at approximately the same time every morning between 9:00 and 12:00. Urine spots with diameters ranging from 0.6 to 5.6 cm was recorded, and others that were larger or smaller were excluded.

### Statistical analysis

Numerical data are presented as mean ± SEM. Statistical analysis of the data sets was carried out using Graphpad Prism version 8.0 (Graphpad software, La Jolla, CA, United States). To compare the expression level of 5-HT_4_R in different layers of the bladder, mean immunofluorescence was quantified. To assess the effects of 5-HT agonist or antagonist on bladder mechanosensory activity, the increase of bladder afferent nerve discharge was plotted against the increase of intravesical pressure for each bladder distension. The averaged area under the curves (AUCs) of the pressure-afferent nerve relationship curves were then compared either using Student’s t tests (to compare two groups) or one-way or two-way ANOVA with appropriate post-tests (to compare multiple groups). Statistical significance was reported at levels of *p* < 0.05 (*), *p* < 0.01 (**), *p* < 0.001 (***) and *p* < 0.0001 (****). The specific tests used for the analysis of each dataset including *p* values, and the sample number (N) are indicated within Results and the individual figure legends.

### Chemicals

RS67333 (Tocris, #0989) and GR125487 (Tocris, #1658) were dissolved in DMSO as stock solution (RS67333, 10 mg/ml, GR125487, 20 mg/ml) and were diluted in isotonic saline (NaCl, 0.9%) to the required concentrations before using.

## Results

### 5-HT_4_R is localized in the murine bladder uroepithelium

**FIGURE 1:**
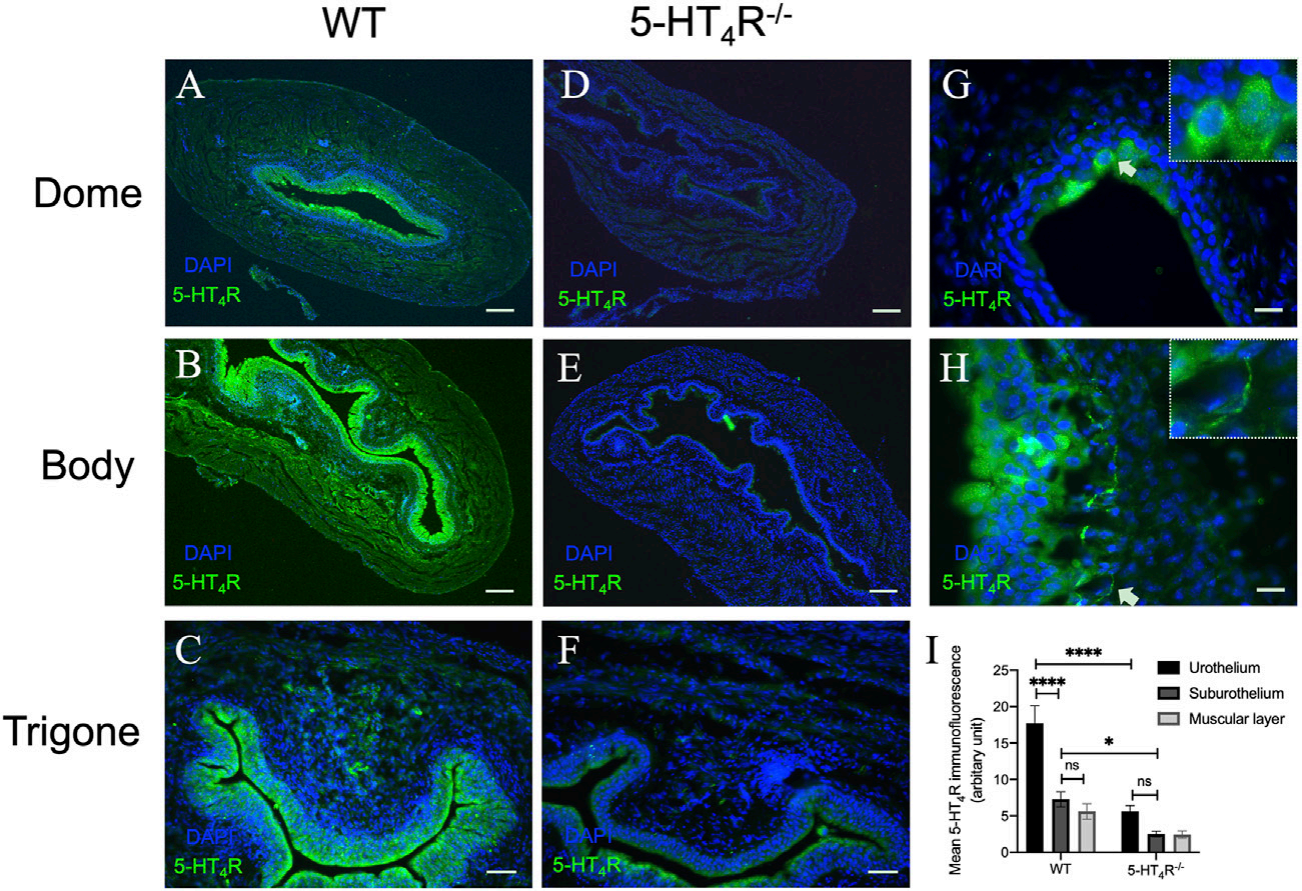
Representative microphotographs of 5-HT_4_R immunofluorescence in murine bladder in WT and 5-HT_4_R^−/−^ mice. All panels are stained with 5-HT_4_R (green) and nuclei are stained by DAPI (blue). 5-HT_4_R immunofluorescence staining at Dome **(A)**, Body **(B)** and Trigone **(C)** of the bladder in WT mice. Specificity of antibody for 5-HT_4_R was confirmed by diminished immunofluorescence in 5-HT_4_R^−/−^ mice **(D–F)**. **(G,H)** High-magnification view of 5-HT_4_R immunofluorescence in WT bladder wall. Arrows indicate strong 5-HT_4_R immunoreactivity in a population of epithelial cells and nerve fibers in subepithelial layer. **(I)** Quantitative analysis of the expression of 5-HT_4_R in different layers of bladder in WT and 5-HT_4_R^−/−^ mice (Two-way ANOVA, *n* = 6 tissue sections from two mice for each group). Calibration bars, 200 μm in **(ABDE)**, 50 μm in **(CF)**, 25 μm in **(GH)**.

To observe the distribution of 5-HT_4_R in the murine urinary bladder, immunofluorescent staining was performed on the bladder tissue sections from WT and 5-HT_4_R^−/−^ mice. Strong 5-HT_4_R immunofluorescence was detected in the uroepithelium of WT but not 5-HT_4_R^−/−^ mice (Figures 1A–F). In the bladder wall of WT mice, interestingly, 5-HT_4_R immunofluorescence was not evenly distributed within the uroepithelium. A proportion of superficial epithelial cells appear to express higher level of 5-HT_4_R immunoreactivity than others (Figure 1G). 5-HT_4_R immunofluorescence was also detected in nerve fibers in the subepithelial layer and in the muscle layer (Figure 1H). Overall, however, 5-HT_4_R immunofluorescence was much more abundant in the uroepithelium than in the subepithelium and the muscle layers (Figure 1I).

**FIGURE 2:**
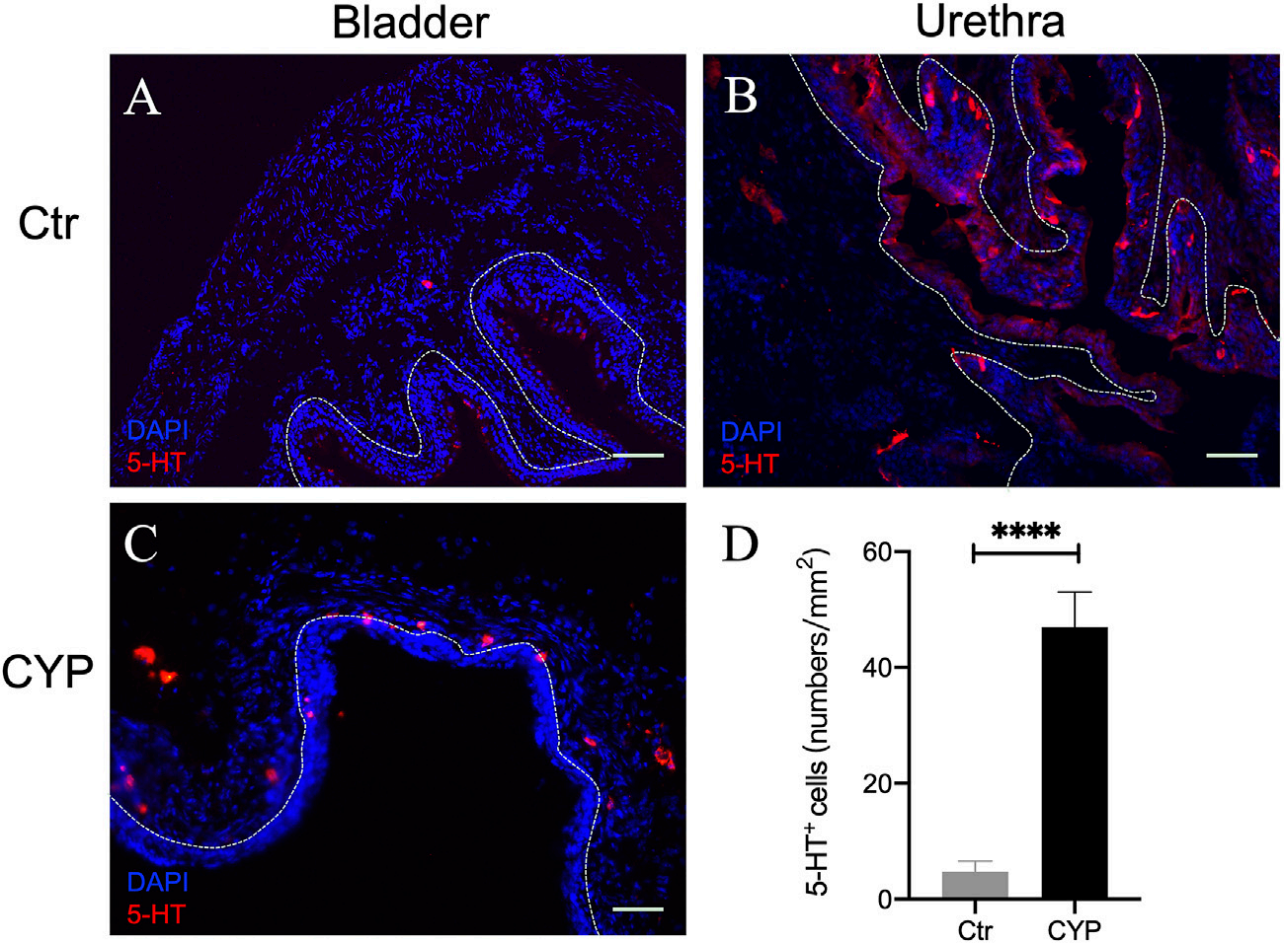
5-HT immunofluorescence in murine bladder and urethra. **(A,B)** show representative 5-HT immunofluorescence (red) in murine bladder and urethra in the control mice, respectively. Note numerous 5-HT immunoreactive neuroendocrine cells were present in urethral but not bladder urothelium. Very few numbers of 5-HT-ir cells (likely mast cells) were encountered in subepithelium in normal bladder. **(C)** Immunofluorescence of 5-HT in the bladder of CYP-treated mice. **(D)** Quantitative analysis of 5-HT positive cells in control and CYP-treated mice. (Unpaired *t* test, *n* = 6 tissue sections from two mice for each group). Dotted white line indicates the boundary of urothelium and lamina propria. Calibration bars, 100 μm in **(A)** and 50 μm in **(B,C)**.

We also carried out immunostaining for 5-HT in the murine urinary bladder and urethra. Interestingly, 5-HT^+^ cells were not detected in the bladder epithelium (Figure 2A), although as expected (Yokoyama et al., 2017; Kullmann et al., 2018), numerous 5-HT^+^ endocrine cells were present in the urethral epithelium (Figure 2B). Very few 5-HT^+^ cells were present in the suburothelium. We suspect those might be mast cells. Consistent with previous reports which showed increased number of mast cells in pathological bladder (Michishita et al., 2015), we found that 5-HT immunoreactive cells in the subepithelium was increased in the CYP-treated mice (Figures 2C,D).

### 5-HT_4_R agonists RS67333 attenuates pelvic afferent response to bladder filling

**FIGURE 3:**
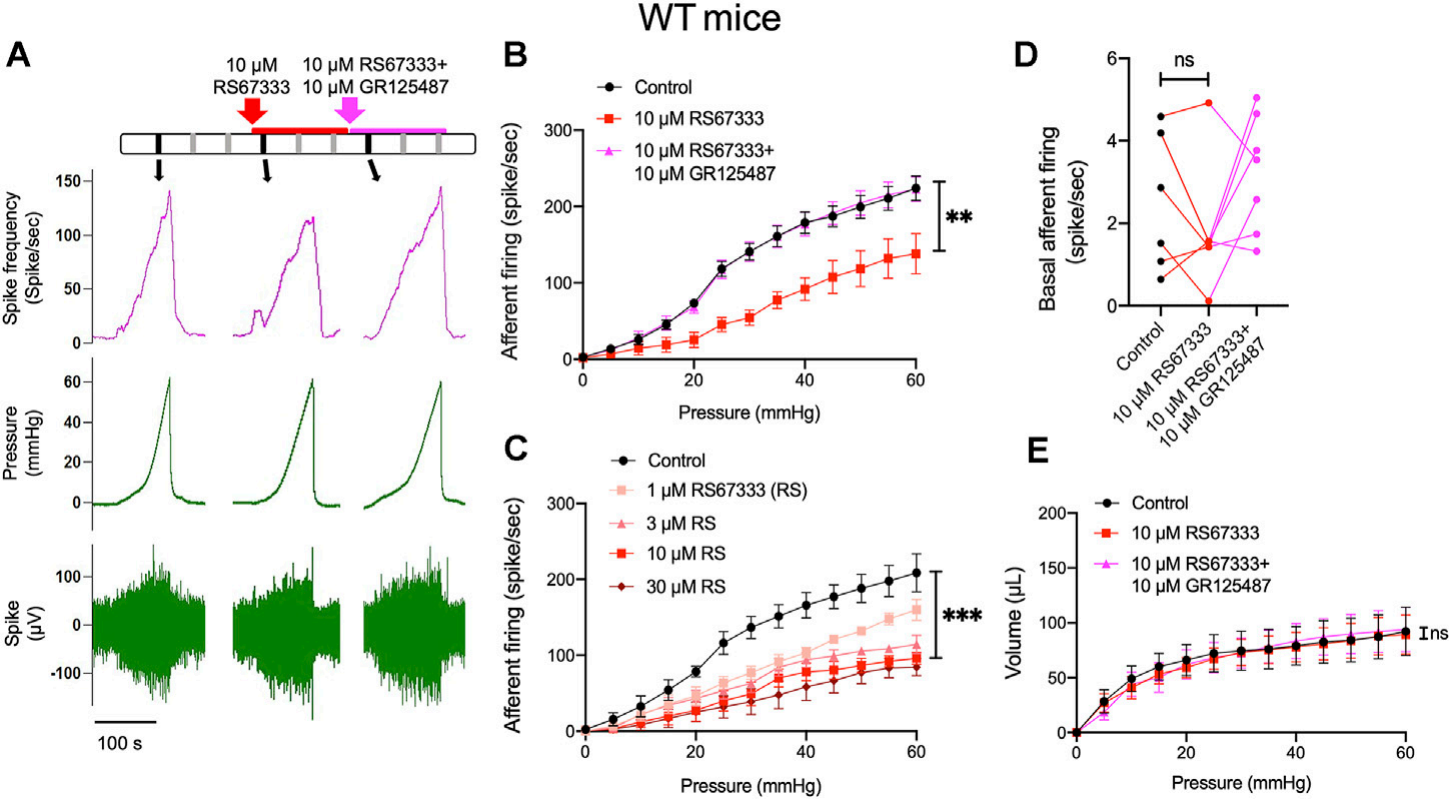
Activation of 5-HT_4_R inhibits mechanosensory activity of bladder afferent nerves. **(A)** Schematic of drug administration and original recordings from an *ex vivo* pelvic nerve recording preparation showing bladder afferent firing and intravesical pressure. Each vertical line represents a distension and the original record of the black vertical lines presented in the below. **(B)** Relationship between bladder afferent firing and intravesical pressure showing a significant decrease in afferent firing in the presence of RS67333 (10 μm) and reversal of the effect by GR125487. The averaged area under the curve (AUC) of Control (1,368 ± 80.8), RS67333 (762.3 ± 130.8), R&G (1,376 ± 77.38) were compared using one-way ANOVA. *n* = 6–8 mice for each group. (**, *p* = 0.002, RS67333 vs. Control.) **(C)** Effects of different concentrations of RS67333 (1, 3, 10 and 30 μm) on the mechanosensitivity of bladder afferent nerves. AUC analysis using one-way ANOVA: Control 1,319 ± 90.14; 1 μM 925.5 ± 53.10 (*p* = 0.0244 vs. control); 3 μm 768 ± 79.09 (*p* = 0.0029 vs. control); 10 μm 607 ± 112.7 (*p* = 0.0012 vs. control); 30 μm 498.1 ± 143.9 (*p* = 0.0003 vs. control). *n* = 4 mice for each group. **(D)** Effect of RS67333 and R&G (10 μm) on basal afferent firing (ns = not significant, one-way ANOVA, *p* > 0.05). **(E)** The relationship between intravesical pressure and volume (the compliance) during ramp bladder distension was unaffected by intravesical administration of RS67333 and R&G (10 μm, ns = not significant, one-way ANOVA, *p* > 0.05).

In order to determine whether 5-HT_4_R have a functional role in bladder sensory signaling, we performed *ex vivo* pelvic nerve recordings to observe the effects of intravesical application of 5-HT_4_R agonist RS67333 on the afferent response to bladder distension. Figure 3A shows a representative experiment in which afferent responses to sequential bladder distensions with saline, saline containing 10 μm RS67333 and saline containing 10 μm RS67333 + 10 μm GR125487 (5-HT_4_R antagonist) were observed. It was evident that RS67333 caused an inhibition of the afferent response to bladder distension and 5-HT_4_R antagonism (with GR123487) reversed this effect (Figure 3B). The inhibitory effect of RS67333 on the afferent response to bladder filling was concentration-dependent (Figure 3C). In this experimental model, bladder afferent nerves had little spontaneous activity before filling (Rong et al., 2002), which was not altered by the 5-HT_4_R agonist (Figure 3D). RS67333 did not significantly affect the pressure-volume curve during bladder filling, indicating that the inhibitory effect of RS67333 on the afferent mechanosensitivity was not due to altered compliance (Figure 3E).

**FIGURE 4:**
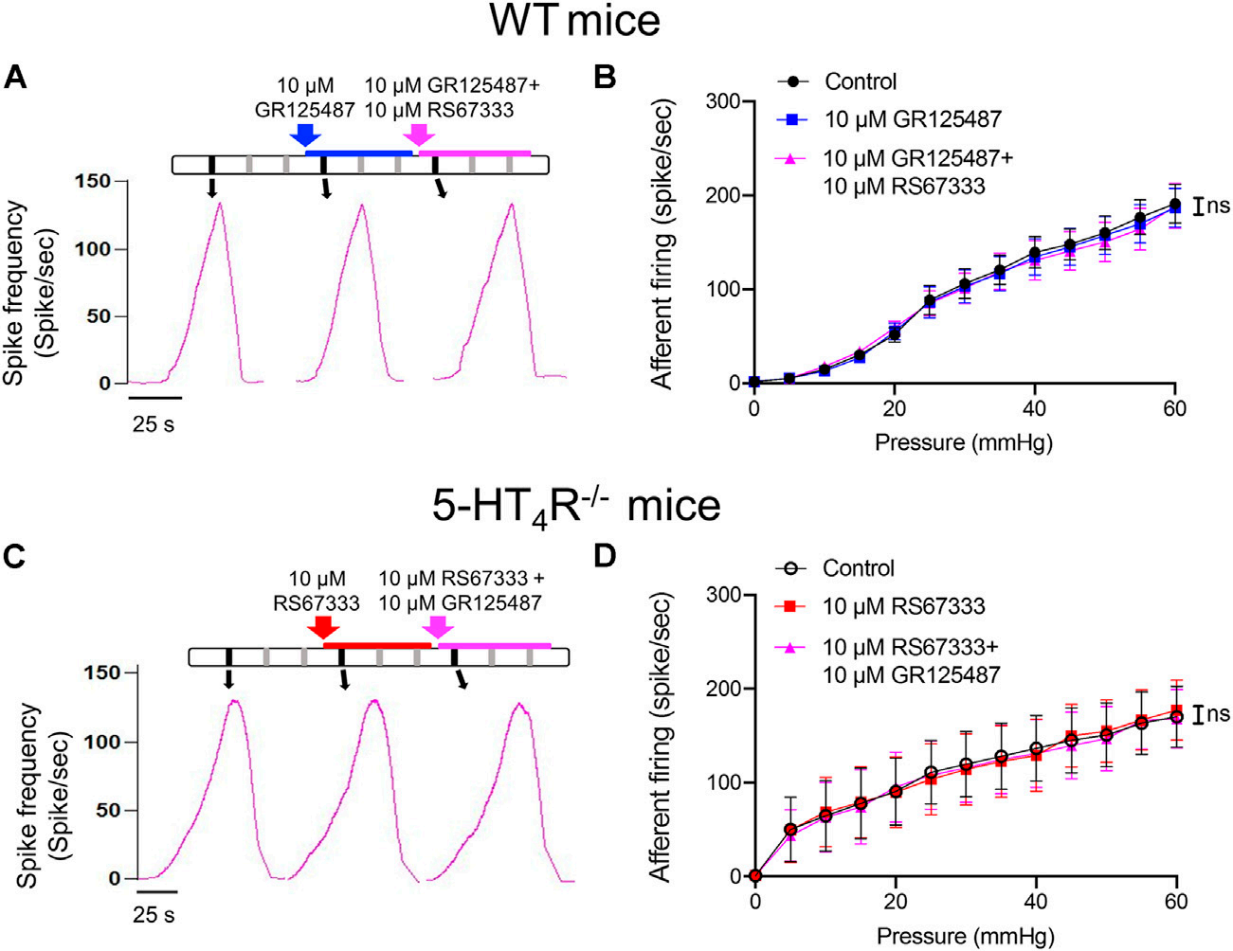
RS67333 had no effect on the mechanosensory responses of the bladder afferent nerves in the presence of 5-HT_4_R antagonist and in 5-HT_4_R^−/−^ mice. **(A)** Schematic of drug administration and the afferent discharge rate during repetitive bladder distensions in an *ex vivo* bladder preparation from a WT mouse. **(B)** Relationship between bladder afferent firing and intravesical pressure (ns = not significant, one-way ANOVA, *p* > 0.05). **(C)** Schematic of drug administration and the afferent firing rate during repetitive bladder distensions in an *ex vivo* bladder preparation from a 5-HT_4_R^−/−^ mouse. **(D)** Relationship between bladder afferent firing and intravesical pressure (ns = not significant, one-way ANOVA, *p* > 0.05). *n* = 4–5 mice for each group.

Further experiments were conducted to determine that the inhibitory effects of RS67333 on the mechanosensitivity were indeed mediated via activation of 5-HT_4_R. In the set of experiment illustrated in Figure 4A, after stable mechanosensory responses were obtained, the bladder was first distended with saline containing 5-HT_4_R antagonist GR123487 and then with GR123487 + RS67333. Neither GR123487 nor GR123487 + RS67333 significantly affected the afferent response to distension (Figure 4B). Furthermore, RS67333 failed to cause an inhibition of the bladder afferent mechanosensory responses in preparations from the 5-HT_4_R^−/−^ mice (Figures 4C,D).

### 5-HT_4_R deficiency exacerbates bladder hypersensitivity in CYP-induced cystitis

**FIGURE 5:**
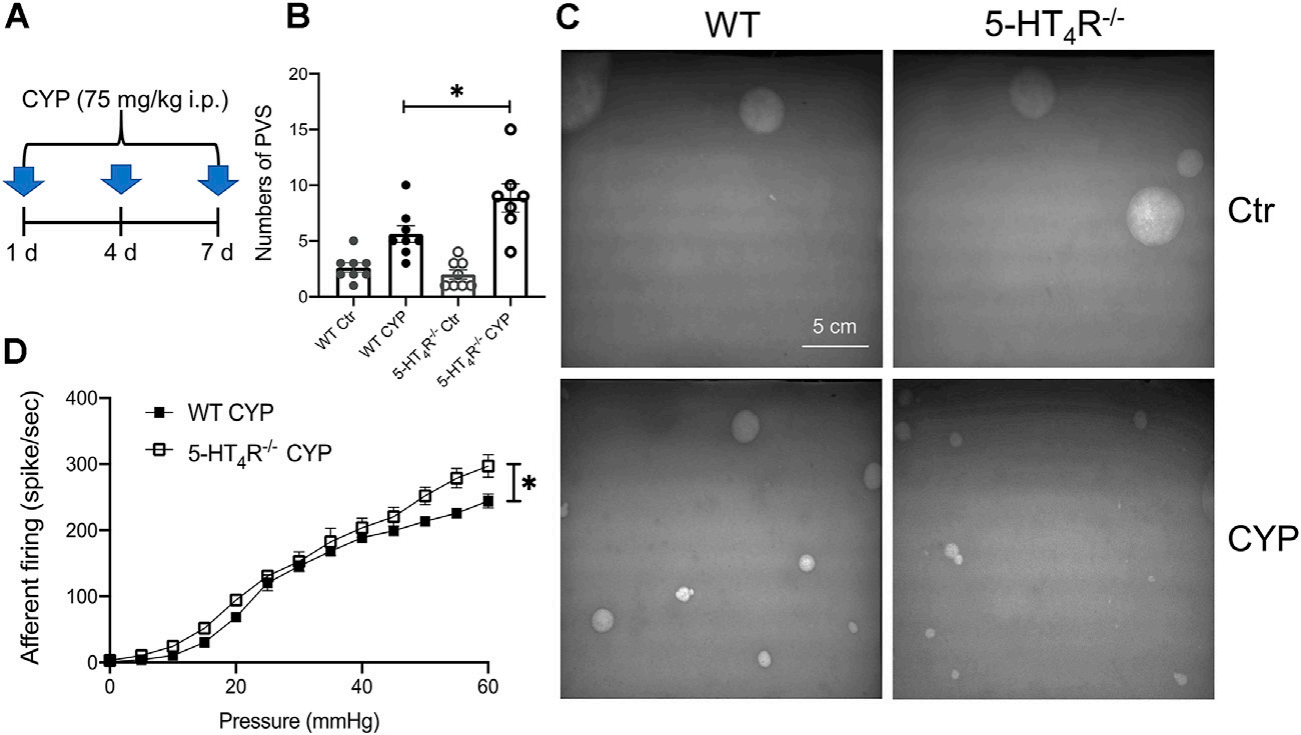
5-HT_4_R deficiency exacerbates bladder hypersensitivity following CYP-induced cystitis. **(A)** Schematic to show protocol of cyclophosphamide (CYP) treatment in WT and 5-HT_4_R^−/−^ mice. **(B)** Statistics on the number of primary void spot (PVS) (Two-way ANOVA, *p* = 0.029, *n* = 7–8 mice for each group). **(C)** Photos of the typical original recordings of void spots. **(D)** Comparison of the pressure-bladder afferent firing relationship curves in the *ex vivo* bladder preparations from CYP-treated WT and 5-HT_4_R^−/−^ mice (AUC analysis: WT CYP 1498 ± 28.28, 5-HT_4_R^−/−^ CYP 1752 ± 84.89, unpaired *t* test, *p* = 0.0219, *n* = 5 mice for each group).

The results so far suggest that uroepithelial 5-HT_4_R mediates an inhibitory effect on mechanosensation of the bladder. We then wondered whether 5-HT_4_R-mediated signaling plays a role in regulation of the mechanosensory activity of inflamed bladder. To address this question, WT and 5-HT_4_R^−/−^ mice were treated with cyclophosphamide (CYP) to induce cystitis (Figure 5A). In the void spot assay, CYP-treated WT and 5-HT_4_R^−/−^ mice both exhibited bladder overactivity with increased urination frequency as compared with their saline-treated controls. Importantly, CYP-treated 5-HT_4_R^−/−^ mice urinated more frequently than CYP-treated WT mice (Figures 5B,C). Consistent with this, the mechanical sensitivity of the bladder afferent nerve in 5-HT_4_R^−/−^ mice was significantly higher than that in WT mice (Figure 5D). Urination frequency was not significantly different between saline-treated WT and 5-HT_4_R^−/−^ mice.

## Discussion

The bladder urothelium is not only a physical barrier that separates the tissue from the urine, but also plays important sensory roles (Birder and Andersson, 2013; Marshall et al., 2020; Vanneste et al., 2021). The urothelium is equipped with a wide array of G Protein-Coupled Receptors (GPCRs), ion channels and transporters, enabling uroepithelial cells to respond to mechanical stretch and luminal contents and to release excitatory and inhibitory mediators, which in turn modulate the activity of bladder afferents (Marshall et al., 2020; Wu et al., 2021). Urothelial sensory transduction participates the regulation of storage and evacuation of urine in normal and disease states. The current study has revealed prominent expression of 5-HT4 receptor in the uroepithelium, which mediates inhibition of mechanosensory activity of bladder afferents. Such mechanism appears to be of physiological relevance since loss of 5-HT_4_R-mediated inhibition exacerbates bladder overactivity following inflammation.

5-HT is a well-known neurotransmitter in the central nervous system. However, the great majority of total 5-HT in the body is synthesized in the periphery (Gershon and Tack, 2007). It is important to understand how peripheral 5-HT affects urinary bladder function since drugs increasing 5-HT bioavailability (such as amitriptyline) were commonly prescribed for the treatment of voiding dysfunctions (Garba et al., 2022) and that serum 5-HT levels appear to be negatively correlated with symptoms of bladder overactivity (Okamoto et al., 2021). 5-HT has been shown to modulate detrusor contraction in different species, acting either prejunctionally (ie, on parasympathetic or sympathetic efferent) or postjunctionally (i.e., on smooth muscles) (Matsumoto-Miyai et al., 2015). We and others have shown in the *ex vivo* bladder preparations that intravesical or bath application of 5-HT causes activation of bladder afferents (Sun et al., 2010; Konthapakdee et al., 2019). These observations show peripheral 5-HT may affect the sensorimotor functions of the urinary bladder.

The impact of 5-HT on urinary bladder functions may be complicated due to the presence of multiple 5-HT receptors. In the urothelium, Konthapakdee et al. detected mRNA for 5-HT1A (1A), 1B, 1D, 2A, 2B, 4, 6, and 7 receptors but not 1F, 2C, 3A, 3B, 5A, and 5B receptors (Konthapakdee et al., 2019), whilst Matsumoto-Miyai et al. found 5-HT1D and 5-HT4 receptors are the predominant 5-HT receptors expressed in murine bladder urothelium (Matsumoto-Miyai et al., 2016). In the current study, by comparing 5-HT_4_R immunofluorescence in WT and 5-HT_4_R^−/−^ bladder, we were able to confirm 5-HT_4_R is highly enriched in murine uroepithelium. Although intravesical administration of 5-HT caused activation of bladder afferents likely through activation of 5-HT_3_R on afferent terminals (Sun et al., 2010; Konthapakdee et al., 2019). We found intravesical instillation of the selective 5-HT_4_R agonist RS67333 caused inhibition of distension-induced bladder afferent discharge. We were confident that the effect of RS67333 was mediated via activation of 5-HT_4_R, since the effect was blocked by the selective 5-HT_4_R antagonists, GR125487, and additionally was absent in the 5-HT_4_R^−/−^ mice. This result was somewhat surprising, since 5-HT_4_R is Gs-coupled and its activation leads to facilitation of transmitter release (Dopamine, Ach, ATP and 5-HT) (Matsumoto-Miyai et al., 2016), inhibition of K^+^ channel (Fagni et al., 1992) and activation of L-type Ca^2+^ channels (Ouadid et al., 1992). It seems possible that activation of the urothelial 5-HT_4_R may facilitate the release of inhibitory mediators such as nitric oxide (NO) and acetylcholine (ACh), which in turn inhibit the excitability of bladder afferent terminals (Consolo et al., 1994; Daly et al., 2010; Yu and de Groat, 2013).

It may be interesting to compare the effects of 5-HT1D and 5-HT4 receptors activation on mechanosensory transduction in the bladder, since both are enriched in the uroepithelium (Matsumoto-Miyai et al., 2016). Matsumoto-Miyai et al. (2016) reported that application of 5-HT to the mucosal side caused inhibition of stretch-induced ATP release and such effect was dependent on 5-HT_1D_R but not 5-HT_4_R. ATP has been the most extensively studied signaling molecule released by the urothelium in response to mechanical stretch or chemical cues such as bacterial lipopolysaccharides (LPS) (Ueda et al., 2020). Multiple P2 purinergic receptors have been detected in the bladder, which may mediate diverse effects of extracellular ATP. Acting on the ionotropic P2X3 or P2X2/3 receptors on sensory terminals, ATP may activate or sensitize low and high threshold bladder afferents (Rong et al., 2002). Conceivably, activation of uroepithelial 5-HT1D receptor would attenuate the mechanosensory responses of bladder afferents due to decreased ATP release in response to mechanical stretch. Therefore, peripheral 5-HT seems to exert dual effects on sensory transductions in the bladder, direct excitatory effect through 5-HT_3_R on sensory neurons and indirect inhibitory effects through 5-HT_1D_R and 5-HT_4_R on uroepithelial cells. Uroepithelial cells are heterogeneous (Cheng et al., 2021). Whether 5-HT_1D_R and 5-HT_4_R are differentially expressed in different populations of uroepithelial cells is an interesting question that needs further investigation. Nevertheless, we noticed that 5-HT_4_R immunofluorescence is not evenly distributed in urothelium. Instead, some superficial urothelial cells seem to express higher level of 5-HT_4_R immunoreactivity than others, which is in favor of the possibility of specific expression in subpopulations of uroepithelial cells. 5-HT_4_R also appears to be expressed on subepithelial and intramuscular nerve terminals. However, neural 5-HT_4_R was unlikely to be involved in the inhibitory effects of intravesical RS67333, since direct activation of the Gs-coupled 5-HT_4_R on sensory terminals would be expected to cause excitatory effects on the afferent activity.

The functional significance of 5-HT_4_R-mediated inhibition of mechanosensory transduction is evident, since loss of 5-HT_4_R exacerbated afferent hypersensitivity and symptoms of bladder overactivity following cyclophosphamide-induced cystitis. We therefore were interested in the endogenous sources of 5-HT to be sensed by uroepithelial 5-HT_4_R. Conceivably, uroepithelial 5-HT_4_R may be stimulated by circulating 5-HT, luminal 5-HT or locally produced 5-HT. Transcripts of the 5-HT synthesizing enzyme, tryptophan hydroxylase 1 (TPH1) is detectable in urothelium (Konthapakdee et al., 2019), although it is still unknown whether uroepithelial cells themselves synthesize and release 5-HT. Akin to enterochromaffin cells in the gut, which are the primary source of circulating 5-HT, 5-HT-releasing neuroendocrine cells are also present in urethra (Kullmann et al., 2018), but have not been reported in the bladder. Mast cells are known to synthesize, store and release 5-HT through degranulation. Previous studies have shown presence of mast cells in the bladder of different species and the number of mast cells reportedly was increased following inflammation or in partial bladder outlet obstruction (pBOO) rat bladders (Michishita et al., 2015). We conducted 5-HT immunofluorescent staining of the murine bladder and found very few 5-HT^+^ cells in the uninflamed bladder. In contrast, 5-HT^+^ neuroendocrine cells were numerous in urethra which was consistent with the previous report (Kullmann et al., 2018). We suspect that in the uninflamed bladder, uroepithelial 5-HT_4_R likely sense circulating and luminal 5-HT. Circulating 5-HT is freely filtered to the renal tubule, so urine content of 5-HT would be determined by circulating 5-HT level and the amount reabsorbed in the renal tubule. A recent community-based study in Japan found that serum 5-HT levels in patients with overactive bladder (OAB) were significantly lower than those in the non-OAB group (Okamoto et al., 2021). We suspect insufficient 5-HT_1D_R or 5-HT_4_R-mediated inhibition of urothelial mechanosensory transduction due to lower serum and luminal 5-HT levels might underlie bladder overactivity in OAB patients. Not surprisingly, we found increased number of 5-HT positive cells (likely mast cells) in the suburothelium of CYP-treated mice. 5-HT derived from those cells may not only interact with 5-HT_3_R on adjacent sensory terminals to directly enhance afferent activity, but also activate the uroepithelial 5-HT_1D_R or 5-HT_4_R to affect urothelial release of mediators which indirectly down-regulate afferent sensitivity. Urothelial 5-HT_1D_R and 5-HT_4_R-mediated inhibition may represent important endogenous protective mechanisms against hypersensitivity following bladder inflammation, as we found 5-HT4R^−/−^ mice exhibited exaggerated bladder overactivity and afferent hypersensitivity in comparison with the WT mice following CYP-induced cystitis.

In summary, the present study has revealed abundant 5-HT_4_R on uroepithelial cells, which mediates inhibition of mechanosensory transduction. Loss of 5-HT_4_R-mediated inhibition may enhance bladder afferent sensitivity and exacerbate bladder over-activity in the inflamed condition. However, the current study has some important limitations that warrant consideration. Firstly, global 5-HT_4_R knockout mice were used in this study, which preclude a definitive conclusion about the functional significance of uroepithelial 5-HT_4_R. Future studies may utilize urothelial specific 5-HT_4_R knockout animal models. Secondly, 5-HT_4_R agonist and antagonist were delivered intravesically to interact with the uroepithelial 5-HT_4_R. Whether they might access beyond the uroepithelium to interact with 5-HT_4_R in the subepithelial and the muscle layer was unknown. To circumvent this, future studies may look at the effects of 5-HT_4_R agonists and antagonists on stretch-induced release of excitatory and inhibitory mediators (eg, ATP, ACh, NO, etc) in mucosal or urothelial cell preparations. Thirdly, in the *ex vivo* bladder preparation, the bladder was distended to 60 mmHg at a filling rate of 0.3 ml/min, which were much higher than the physiological filling rate and pressure. Fast filling rate is advantageous when the *ex vivo* bladder needs to be distended repetitively for prolonged experimental protocol, but may favor the activation of high threshold afferent fibers. A slower filling rate may be preferred to study the influence of 5-HT4R on urothelial sensory transduction pertinent to the storage and evacuation of urine in physiological conditions. Despite the limitations, the current study has provided the first evidence that 5-HT_4_R may be involved in regulation of the urothelial sensory function and may potentially be targeted for the treatment of bladder overactivity and pain.

## Data Availability

The original contributions presented in the study are included in the article/Supplementary Material, further inquiries can be directed to the corresponding author.
